# Computational scanning tunneling microscope image database

**DOI:** 10.1038/s41597-021-00824-y

**Published:** 2021-02-11

**Authors:** Kamal Choudhary, Kevin F. Garrity, Charles Camp, Sergei V. Kalinin, Rama Vasudevan, Maxim Ziatdinov, Francesca Tavazza

**Affiliations:** 1 Material Measurement Laboratory, National Institute of Standards and Technology, Gaithersburg, MD 20899 USA; 2 Center for Nanophase Materials Sciences, Oak Ridge National Laboratory, Oak Ridge, TN 37831 USA

**Keywords:** Electronic structure, Surfaces, interfaces and thin films

## Abstract

We introduce the systematic database of scanning tunneling microscope (STM) images obtained using density functional theory (DFT) for two-dimensional (2D) materials, calculated using the Tersoff-Hamann method. It currently contains data for 716 exfoliable 2D materials. Examples of the five possible Bravais lattice types for 2D materials and their Fourier-transforms are discussed. All the computational STM images generated in this work are made available on the JARVIS-STM website (https://jarvis.nist.gov/jarvisstm). We find excellent qualitative agreement between the computational and experimental STM images for selected materials. As a first example application of this database, we train a convolution neural network model to identify the Bravais lattice from the STM images. We believe the model can aid high-throughput experimental data analysis. These computational STM images can directly aid the identification of phases, analyzing defects and lattice-distortions in experimental STM images, as well as be incorporated in the autonomous experiment workflows.

## Abstract (summary)

**Table:** 

Measurement(s)	material entity • 2-dimensional material
Technology Type(s)	Scanning Tunneling Microscopy • density functional theory
Factor Type(s)	exfoliable 2D material

Machine-accessible metadata file describing the reported data: 10.6084/m9.figshare.13573820

## Background & Summary

Since the invention of the scanning tunneling microscope (STM)^1^, this technique has become an essential tool for characterizing material surfaces and adsorbates. In addition to providing atomic insights, STM has been proven useful for characterizing the electronic structure, shapes of molecular orbitals, and vibrational and magnetic excitations^2,3^. It can also be used for manipulating adsorbates and adatoms, and for catalysis and quantum information processing applications^3–8^. Quantum mechanics-based density functional theory (DFT) has often been used to produce virtual STM images for these applications^9,10^. However, a systematic database of such computational STM data is still lacking. As DFT-STM images are constructed using defect-free materials, they provide standard reference images (SRI) that are useful to aid in identifying phases^11,12^, analyzing defects^13,14^ and quantifying lattice-distortions^15^ in experimental STM images. A DFT-STM database is therefore essential to provide a direct link between atomic positions and images, which can aid experimental analysis. Moreover, the orbital-projected electronic density of states available in our database can help explain which atoms and orbitals contribute to an experimental STM image. Finally, a computational database can provide an accurate training set for developing machine learning (ML) models to rapidly analyze experimental STM images.

STM imaging is particularly well-suited to studying two-dimensional (2D) materials, such as graphene^16^, MoS_2_^17^, NbSe_2_^18^, WSe_2_^19^, WTe_2_^20^, FeSe^21^, black-phosphrous^22,23^ and SnSe^24^. 2D materials^25,26^ has opened diverse areas of application, such as sub-micron level electronics^27^, flexible and tunable electronics^28^, superconductivity^29^, photo-voltaics^30^, water-purification^31^, sensors^32^, thermal-management^33^, energy-storage^34^, medicine^35^, quantum dots^36,37^ and composites^38–40^. The surfaces of 2D materials are unique because they lack dangling bonds, allowing them to be exfoliated. This property makes them ideal candidates for building a database of computational STMs images because they don’t require thick slabs perpendicular to the surface, which are computationally expensive to simulate accurately, and they do not have surface reconstructions. The generation of STM images for perfect systems is an initial step, and we will extend this project to include defective systems and the effect of thermal noise in the future.

In this work, we use DFT to generate STM images of exfoliable 2D materials. We use the recently developed JARVIS-DFT database (https://jarvis.nist.gov/jarvisdft) and select 2D materials with exfoliation energy less than 200 meV/atom. The JARVIS-DFT database contains about 40000 bulk and 1000 two-dimensional materials with their DFT-computed structural, energetic^26^, elastic^41^, optoelectronic^42^, thermoelectric^43^, piezoelectric, dielectric, infrared^44^, solar-efficiency^45^, and topological^46,47^ properties. We note that there are several factors that can influence the appearance of experimental or DFT-based STM image predictions, such as the STM-tip material, bias voltage, and the scanning mode, *i.e*. constant-height mode (CHM) vs. constant current mode (CCM). Similarly, there are several methods for simulating STM images using DFT, including Bardeen^48^, Tersoff-Hamman^49^ and Chen^3^ methods. Here, we present results for constant height and constant current DFT-STM images computed using the Tersoff-Hamann approach^49^, which assumes a non-functionalized (*s*-wave) STM tip. Hence, in the simulation we don’t explicitly model the tip and its interactions. The ML model training is based on CHM images. The DFT-STM database currently contains images for 716 exfoliable 2D materials, with additional computations ongoing. All the DFT-STM data will be uploaded into the JARVIS-DFT database.

As a first example application of this database and use artificial intelligence methods^50–53^, we use the computational STM images to train a convolution neural network ML classification model for Bravais-lattices. This model is able to quickly classify STM images into the five lattice classes (square, hexagon, rhombus/centered-rectangle, rectangle and parallelogram/oblique) that are possible for 2D systems. Such classifications are of importance, for example when dealing with phase transitions^54^. They can also be used as an aid to automatic conventional crystallographic image processing of big datasets and to obtain information from noisy images. This work acts as a starting point for identifying the defects in experimental images by providing a collection of ideal STM images for comparison purposes^55^. Ideally one would use an information-theoretic approach, as opposed to deep learning, to enable space group determination with uncertainty quantification, as demonstrated by Moeck, to distinguish the specific subgroups of selected group^56^. However, a pre-screening step can be rapidly accomplished with a suitably trained neural network as shown here, which should then be verified using the approach outlined in ref. ^54^. Later, these computational STM trained models can be integrated with experiments for active learning processes^50,57^.

## Methods

All DFT calculations are carried out with Vienna *ab initio* simulation package (VASP)^58,59^ using projected augmented wave (PAW) formalism and using vdW-DF-OptB88 functional^60^. Note that for monolayers, vdW functionals are not strictly necessary. But we include vdW interactions to be consistent with our JARVIS-DFT 3D dataset. Also, we plan to develop multi-layer materials databases, which do require vdW interactions. The vdW functional works for both strongly and weakly bonded systems^41^. All the machine learning trainings are carried using Keras with TensorFlow backend^61^. Note that commercial software is identified to specify procedures, and such identification does not imply recommendation by the National Institute of Standards and Technology. The k-point and plane-wave cut-off convergence for each material are obtained using the workflow detailed in ref. ^45^. The high-throughput computation and analysis tools will be made available at JARVIS-Tools github page: https://github.com/usnistgov/jarvis. The 2D materials are provided with at least 20 Å vacuum in the *z*-direction to avoid self-interactions. The force and energy convergence for DFT self-consistent calculations are 10^−6^ eV and 0.001 eV/Å respectively.

The STM images are calculated using the Tersoff-Hamann approach, which is a simple model of an *s*-wave STM tip^49^:

1 $$n\left(r,\,E\right)=\sum _{\mu }{\left|{\psi }_{\mu }\left(r\right)\right|}^{2}\delta \left({\varepsilon }_{\mu }-E\right)$$

2 $$I\left(r,\,V\right)\,\propto \,\underset{{E}_{F}}{\overset{{E}_{F}+eV}{\int }}dE\,n(r,\,E)$$

In this approach, the tunneling current *I*, which depends on the tip position *r* and the applied voltage *V*, is proportional to the integrated local density of states (ILDOS). The ILDOS is calculated from the Kohn-sham eigenvectors, ${\psi }_{\mu }$, and eigenvalues, ${\varepsilon }_{\mu }$, where μ labels different states. *E*_*F*_ is the Fermi-energy. Different experiments will choose different applied voltages, but we concentrate on two values, + 0.5 eV for positive bias and −0.5 eV for negative bias, which require integrating from *E*_*F*_ to ${E}_{F}\pm 0.5\,eV$. We choose 0.5 eV range for simplicity sake, and other values usually produce qualitatively similar images for metals or small gap semiconductors. However, simulations for other voltages should also be possible with the method and tools discussed in this work.

This method is readily available in DFT software such as VASP^62^. Please note that plane-wave codes like VASP will not accurately describe the exponential decay of the wave functions far away from the atoms, and wave functions may need to be extrapolated in order for STM simulations at large heights such as 7 Å else it can show unphysical effects^63^. Hence, we choose image height relatively close to surface. All the STM images are made at least 20 Å long in the *xy* plane by repeating the primitive unit cell. We choose height 2 Å above the surface (maximum of z-coordinate) during the simulations. For constant-current images, we identify iso-surfaces that have a constant ILDOS. The height of these iso-surfaces at each *xy*-coordinates produces the images.

For the machine learning model, we simplify the constant-height STM images using a black/white color-scheme and choose a pixel value of 170 (out of maximum 255) for finding atomic features. We simplify the images because the image produced from the wavefunction is still on a continuous scale (i.e. grey image), while for the Bravais lattice classification only requires information on whether an atom is there or not. Based on the lattice-parameters and angles the 2D materials can be classified in five classes: 1) hexagonal, 2) square, 3) rhombus/centered-rectangle, 4) rectangle, 5) parallelograms/oblique. Deep-learning image recognition tasks typically require thousands of training images. To increase the size of our training set, we use several commonly applied image augmentations: random rotations, flipping, zooming in and zooming out. We apply augmentations until all the five classes have at least 10000 images leading 53508 images. Image processing ML models are usually non invariant to the operations mentioned above, which is why the initial dataset is augmented with such operations. We use a multi-layer network with four convolution layers (with 16, 32, 48 and 64 feature-maps and with kernel-size of 3), four max-pooling layers (with pool-size of (2, 2)) activated by a rectified linear unit (ReLU), one fully-connected 600-nodes layer with ReLU activations, and a fully-connected softmax layer with five outputs. Since the entire dataset is too big to feed to the GPU memory at once, we divide it into multiple smaller batches. The total number of training examples present in a single batch (batch size) is 32 for our NN model. We have 20% dropout before the softmax layer to avoid overfitting. We use ADAM stochastic optimization method for gradient descent with ‘sparse categorical crossentropy’ as loss function. We split the dataset into training, validation, and test sets. We use a 90%-10% train-test split for the entire dataset in such a way that both training and testing data have a proportionate amount of all the five classes. Furthermore, we apply a 90%-10% split on the training data for model-training, validation and generating the learning curve. We apply ‘Early-stopping’ to avoid over-fitting of the model. After the model development, we apply this model on the 10% test-data to evaluate the accuracy of the model. Note that the 10% test-dataset was never used during model development.

During the training, we monitor the train-validation curve (discussed later) to avoid overfitting. We use accuracy, precision, recall, and F1-score to measure the overall and individual class performances. The precision is the ratio $\frac{{\rm{TP}}}{{\rm{TP}}+{\rm{FP}}}$ where TP is the number of true positives and FP the number of false positives. The recall is the ratio $\frac{{\rm{TP}}}{{\rm{TP}}+{\rm{FN}}}$ where TP is the number of true positives and FN the number of false negatives. The recall is intuitively the ability of the classifier to find all the positive samples. The F1-score can be interpreted as a weighted harmonic mean of the precision and recall, where an F1-score reaches its best value at 1 and worst score at 0. The overall classification accuracy of the model is given as $\frac{{\rm{TP}}+{\rm{TN}}}{{\rm{TP}}+{\rm{TN}}+{\rm{FP}}+{\rm{FN}}}$, where TN represents the number of true-negatives. We also use the confusion matrix to show the percentage correct and incorrect predictions of each class. Both the model and the associated dataset will be made publicly available soon at the JARVIS-DFT website.

## Data Records

After the calculations, the metadata is stored in the Javascript Object Notation Files (JSON) format which can be easily integrated with databases such as MongoDB. The dataset is made publicly available through the JARVIS-STM (https://jarvis.nist.gov/jarvisstm) web-app. The web-app provides both constant-height and constant-current simulation features and allows the user to change the chosen height or current value. We have made the dataset publicly available through Figshare repository^64^ as well. The dataset consists of positive and negative bias constant-height images in Joint Photographic Experts Group (JPEG) format for the 2D materials under investigation. In addition to the images, we provide the raw input/output files for the calculations (including PARCHG files) at to enhance reproducibility of the work that could be used for generating both constant height and constant current images and for a given size of the xy-dimension.

## Technical Validation

### Validation of DFT simulated images

**Fig. 1:**
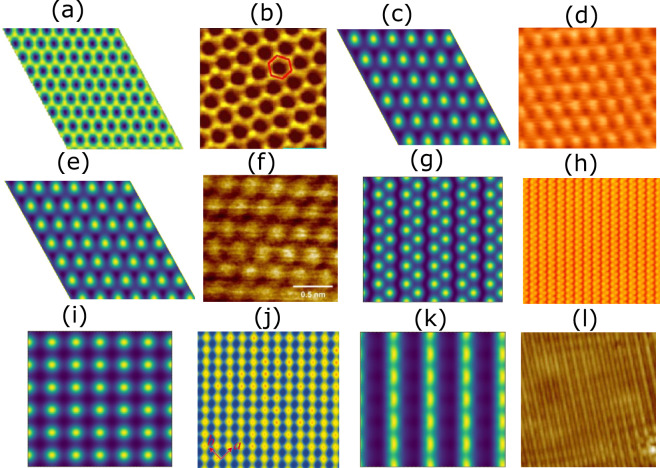
Comparison of computational and experimental STM images. Columns 1 and 3 are computational images while 2 and 4 are corresponding experimental images. (**a**) DFT-STM image of graphene, (**b**) experimental STM image of graphene[Reprinted with permission from ref. ^16^. Copyright 2019 American Physical Society], (**c**) DFT-STM image of 2H-NbSe_2_, (**d**) experimental STM image of 2H-NbSe_2_ [Reprint from ref. ^18^ with the permission of AIP Publishing.], (**e**) DFT-STM image of 2H-MoS_2_, (**f**) experimental STM image of 2H-MoS_2_ [Reprint from ref. ^17^ with the permission of AIP Publishing.], (**g**) DFT-STM image of black phosphorous, (**h**) experimental STM image of black-P [Adapted with permission from ref. ^22^, Copyright 2019 American Physical Society], (**i**) DFT-STM image of FeSe, (**j**) experimental STM image of FeSe [Reprint from ref. ^21^ with the permission of AIP Publishing.], (**k**) DFT-STM image of 1T’-WTe_2_, (**l**) experimental STM image of 1T’-WTe_2_ [Reprinted with permission from ref. ^20^ Copyright 2017 by the American Physical Society].

We simulate computational STM images of 716 exfoliable materials (E_f < _200 meV/atom) using the Tersoff-Hamann approach. We compare computational STM images with those from experiments for graphene^16^, 2H-MoS_2_^17^, 2H-NbSe_2_^18^, 2H-WSe_2_^19^, 1T’-WTe_2_^20^, FeSe^21^, black-P^22,23^, SnSe^24^, Bismuth^64,65^. We chose these systems because we could find well-characterized experimental images in the literature. Qualitatively, we observe that the patterns in the computational and experimental STMs are very similar (see Fig. 1).

Experimental STM images for each system can be found in appropriate reference. Note that we are able to predict the STM for 2D vdW materials very well because they lack dangling bonds. Such images with non-vdW systems such as Si (111)^66^ would require bigger simulation cells in the xy direction to accommodate reconstructions, as well as many additional layers to converge the calculations.

**Fig. 2:**
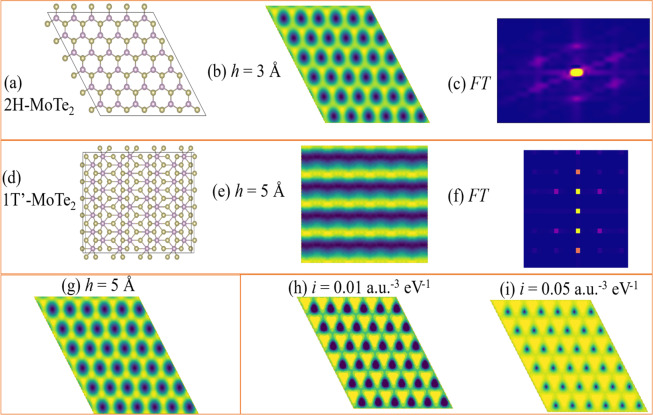
STM images of MoTe_2_ in 2H and 1T’ phases, their fast Fourier transform (FT), height and current dependence. (**a**) crystal structure of 2H-MoTe_2_ (JVASP-670) (**b**) its constant-height STM image at the height of 3 Å, (**c**) FT of the STM image, (**d**) crystal structure of 1T’-MoTe_2_ (JVASP-673) (**e**) its STM image at the height of 5 Å, (**f**) FT of the STM image, (**g**) constant-height STM image of 2H-MoTe_2_ at the height of 5 Å to compare with Fig. b, (**h**) constant current image for 2H-MoTe_2_ at constant current 0.01 a.u.^−3^ eV^−1^, (**i**) constant current image for 2H-MoTe_2_ at constant current 0.05 a.u.^−3^ eV^−1^.

The DFT-STM can be used for distinguishing phases such as the 2D-monolayer 2H-MoTe_2_ (JVASP-670) and 1T’-MoTe_2_ (JVASP-673) phases, as shown in constant height positive bias conditions in Fig. 2. Such phase-identifications can be helpful in providing insight into phase-transformation mechanisms during experiments.

**Fig. 3:**
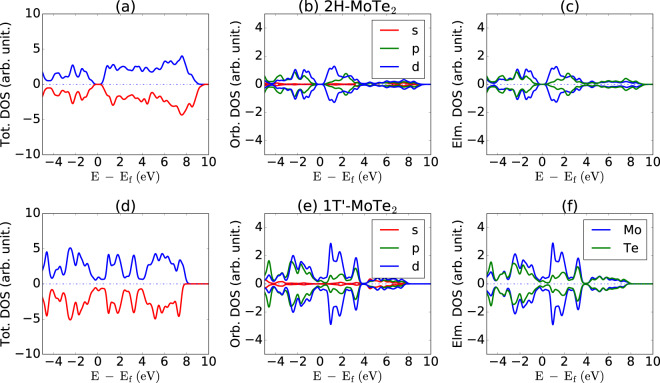
Total (**a**,**d**), element (**b**,**e**) and orbital(**c**,**f**) projected density of states of materials discussed in Fig. 1. The Fermi-energies are set to zero. Total up and down spins are shown as blue and red lines respectively in (**a**, **d**).

The 2H-phase is semiconducting material with hexagonal symmetry, as is evident from the crystal structure in Fig. 2a. The positive + 0.5 eV bias constant height image of this structure is shown in Fig. 2b. The electronic states in this range are dominated by Mo (*d*-orbital) states, hence the brighter spots in the STM are dominated by Mo *d*-orbitals, which can be understood by analyzing the projected density of states (Fig. 3). As shown in Fig. 2c, the fast Fourier transform (FT) of the simulated STM image in Fig. 2b shows hexagonal symmetry. Similarly, the crystal structure, STM image and FT of rectangular 1T’-MoTe_2_ is shown in Fig. 2d–f respectively. We note that the FT of the STM image of a rectangular system with a multi-atom cell is not a simple rectangle. We show examples of variation of height in Å and current in arbitrary units in Fig. 2g,h and i for 2H-MoTe_2_. The constant height for 2H-MoTe_2_ in Fig. 2b is for 3 Å while that in Fig. 2g is for 5 Å with respect to the highest atom in the cell. Clearly, the hexagonal patterns remain the same, but the structure around the atoms slightly changes due to the change in height. This is because as we move the hypothetical STM tip, we probe different layers of charge density. Similarly, we show the current variation based STM images for 0.01 and 0.05 a.u.^−3^ eV^−1^ in Fig. 2h,i. Note that it is difficult to quantitatively compare the computational and experimental STM images because the tunneling-current is critically dependent on the specific experimental setup.

**Fig. 4:**
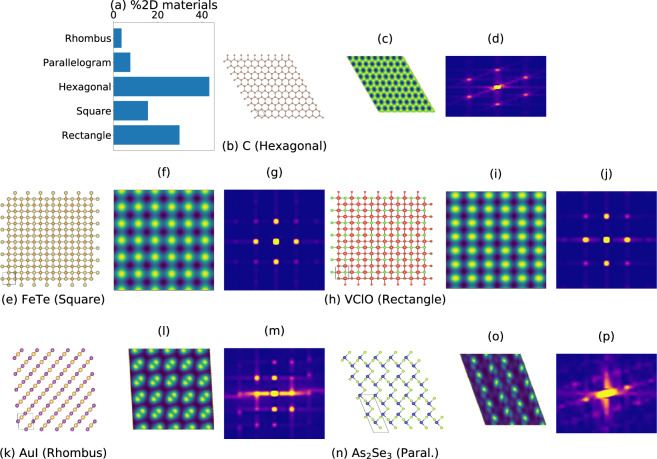
Types of 2D lattices with examples. (**a**) 2D lattice-type distribution in the database. (**b**–**d**) crystal structure, constant height STM (CHS) and FT for graphene. Similar images are also provided for (**e**–**g**) FeTe, (**h**–**j**) VClO, (**k**–**m**) AuI, (**n**–**p**) As_2_Se_3_.

Based on lattice parameter information in 2D plane, the 2D materials lattices can be classified in 5 types: hexagon, square, rectangle, rhombus/centered-rectangle, and parallelograms/oblique. We classify all the 2D materials in our database, with the distribution shown in Fig. 4a. Most of the 2D materials in our database are hexagonal, followed by rectangular and square lattices. In Fig. 4, we give examples of materials in each lattice type, in each case showing the atomic positions, a constant height STM image, and the fast Fourier transform (FT) of the STM image. An example of hexagonal lattice is shown in Fig. 4b graphene (JVASP-667). It is one of the most widely investigated 2D materials. The STM positive bias image for graphene is shown in Fig. 4c. An FT of the image Fig. 4c is shown in Fig. 4d. It is clear from Fig. 4d that there is a hexagonal pattern due to hexagonal symmetry in graphene. Similarly, for the square lattice example, FeTe (JVASP-6667), the crystal structure, STM, and FT are shown in Fig. 4e–g. Fe *d*-states mainly contributes to the STM image in Fig. 4f. The FT of this image shows square-like patterns in Fig. 4g. Similarly, Fig. 4h gives the crystal structure of VClO (JVASP-8933), and its STM and FT show a rectangular pattern (Fig. 4i,j). AuI (JVASP-6187) has a centered-rectangle structure, as shown in Fig. 4k. The lattice constants are 4.274 Å and the angle between them is 93.2 degrees. The Au *d*-orbitals contribute most to the STM image. The atomic and orbital projected density of systems for all the systems here is given in the supplementary information (Supplementary Fig. S1) and the respective webpages for each material. The FT in the Fig. 4m,p shows a noticeable blur, which can be caused by the truncation of the infinite slab to a finite image. Note that the mathematical FT of a perfectly periodic system would have ideal/sharp peaks. However, we purposefully truncate the images and include white spaces to mimic experimental images. Hence, they won’t be perfectly sharp. Figure 4n shows As_2_Se_3_ (JVASP-13544), an example material with an oblique unit cell with lattice constants of 4.4 and 12.9 Å and an angle of 109.9 degrees. The FT of the STM in Fig. 4p is difficult to interpret.

### Machine learning model development

**Fig. 5:**
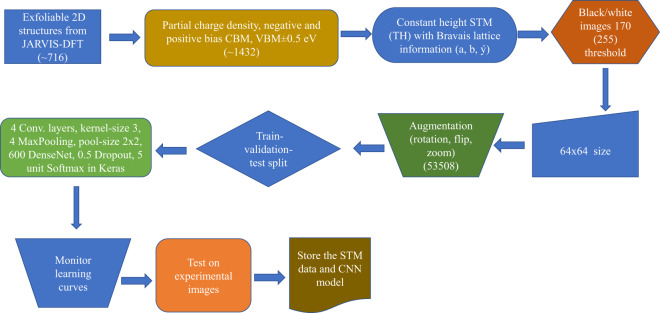
Flow-chart showing the steps involved in the generation of STM-dataset and the machine learning process.

Having prepared our database, we now train a ML model (JARVIS-STMnet) following the flow-chart in Fig. 5.

**Fig. 6:**
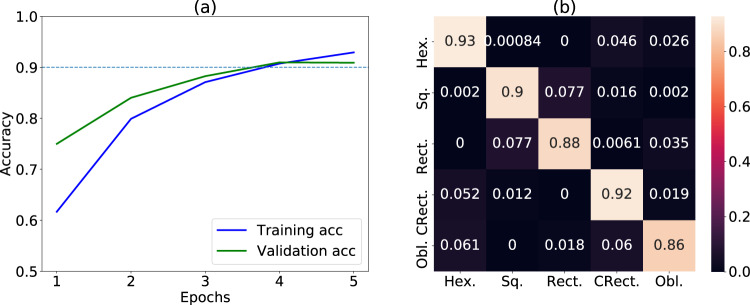
Performance of the machine learning model. (**a**) learning curve, (**b**) confusion matrix.

In Fig. 6 we show the convolution neural network training and the learning curves for the deep learning model. We monitor the learning curve as in Fig. 6a. We see that after the 5^th^ epoch the training and validation accuracy curves begin to diverge, so we stop further training. We obtain 90.1% accuracy on the validation set and 90.0% accuracy on the 10% test-set, which was never used during the training process. The difference between the training and the validation curve is small, implying low overfitting. We apply the trained model on the 10% test-set data and the confusion matrix is shown in Fig. 6b. We also provide precision, recall and F1 scores in Table 1. The baseline accuracy of the model is 1/5 = 20%. Clearly, the overall accuracy is more than 4 times higher than the random-guessing baseline model. Also, all the scores in Table 1 are more than 0.85, indicating that the model performs much better than a random guessing model. Note that although the accuracy is a measure of the overall model, it is important to investigate the prediction accuracy for each class of the model. A confusion matrix with high diagonal element values signifies high accuracy. It is clear from the Fig. 6b that the model performs excellently for hexagonal, centered rectangle and square lattices, and less well for the rectangle and oblique lattice types. Moving beyond simulated STM images, as an initial validation, we apply the model to nine experimental images discussed above for an initial more realistic test step for graphene^16^, 2H-MoS_2_^17^, 2H-NbSe_2_^18^, 2H-WSe_2_^19^, 1T’-WTe_2_^20^, FeSe^21^, black-P^22,23^, SnSe^24^, Bismuth^64,65^. We find that the model predicts the correct class for seven of them. Performing a more systemic analysis of our model’s accuracy on experimental images would require a database of hundreds of experimental images, and such a database is currently not available. We hope this work will spur the development of such a database. Also, as we make the entire dataset publicly available, and we hope that other researchers could apply their machine-learning models on this dataset.

**Table 1:** Classification report of classifying 2D constant-height STM images into lattice-types.

Lattice	Precision	Recall	F1-score
Hexagonal	0.91	0.93	0.92
Square	0.90	0.90	0.90
Rectangle	0.91	0.88	0.90
Centered Rectangle	0.86	0.92	0.89
Oblique	0.91	0.86	0.88
Accuracy			0.90

## Usage Notes

We introduce the first systematic database of scanning tunneling microscope (STM) images obtained using density functional theory (DFT) for two-dimensional (2D) materials. Specifically, the database is constructed using the Tersoff-Hamann method for constant-height images. Although only defect free materials are considered in this work, STM image dataset with defects will be developed soon. We anticipate that this dataset and methods used will provide a useful tool in fundamental and application-related studies of materials. Experimental verification provides insight into understanding the applicability and limitation of our DFT data. Based on the list of data, the user will be able to choose particular materials for specific applications. Data mining, data analytics, and artificial-intelligence tools then can be added to guide screening of materials.

## Supplementary information

Supplementary Information

## Data Availability

Python-language-based codes with examples are given at the JARVIS-Tools page https://github.com/usnistgov/jarvis.
