# Supplementary material for: Computational scanning tunneling microscope image database
Source: Sci Data. 2021 Feb 11;8:57. doi: 10.1038/s41597-021-00824-y (PMC7878481; doi:10.1038/s41597-021-00824-y)
Supplement: Supplementary file 1 — Supplementary Information [file 41597_2021_824_MOESM1_ESM.docx]

**Supplementary info:** **Computational scanning tunneling microscope image database**

Kamal Choudhary^1^, Kevin F. Garrity^1^, Charles Camp^1^, Sergei V. Kalinin^2^**,** Rama Vasudevan^2^, Maxim Ziatdinov^2^, Francesca Tavazza^1^

1. Material Measurement Laboratory, National Institute of Standards and Technology, Gaithersburg, MD 20899, USA.

2. Center for Nanophase Materials Sciences, Oak Ridge National Laboratory, Oak Ridge TN 37831, USA.


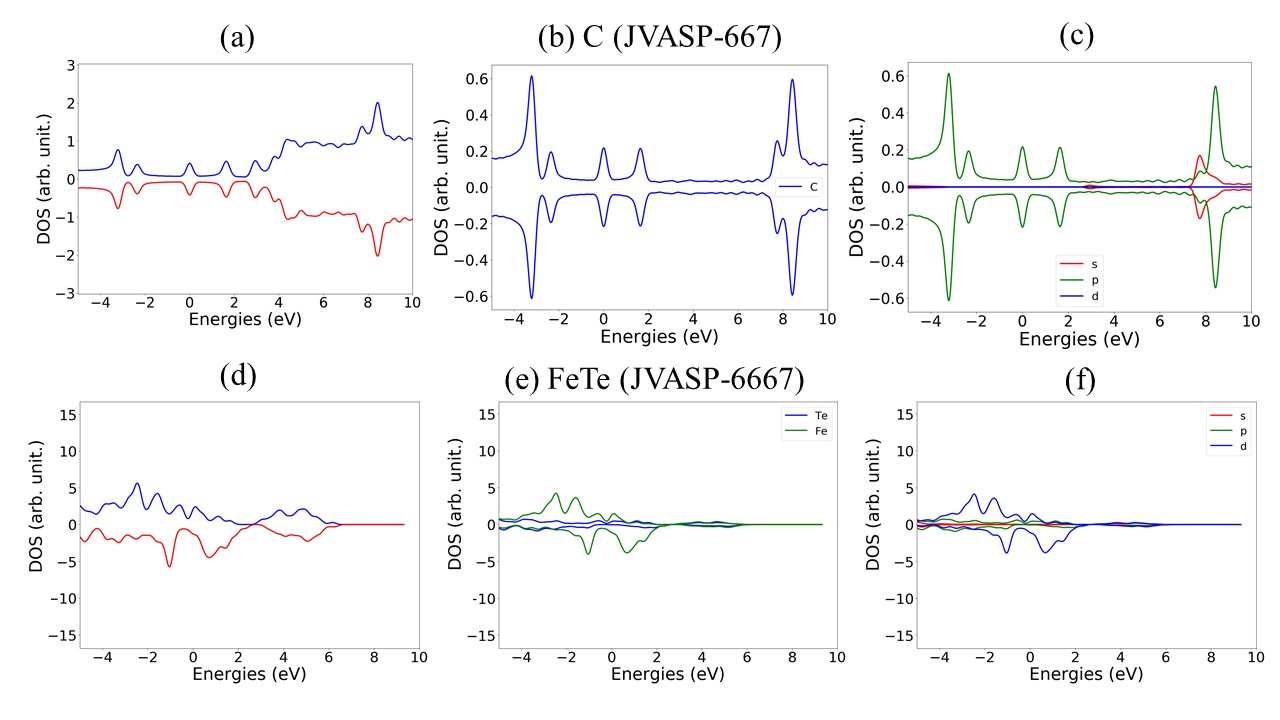


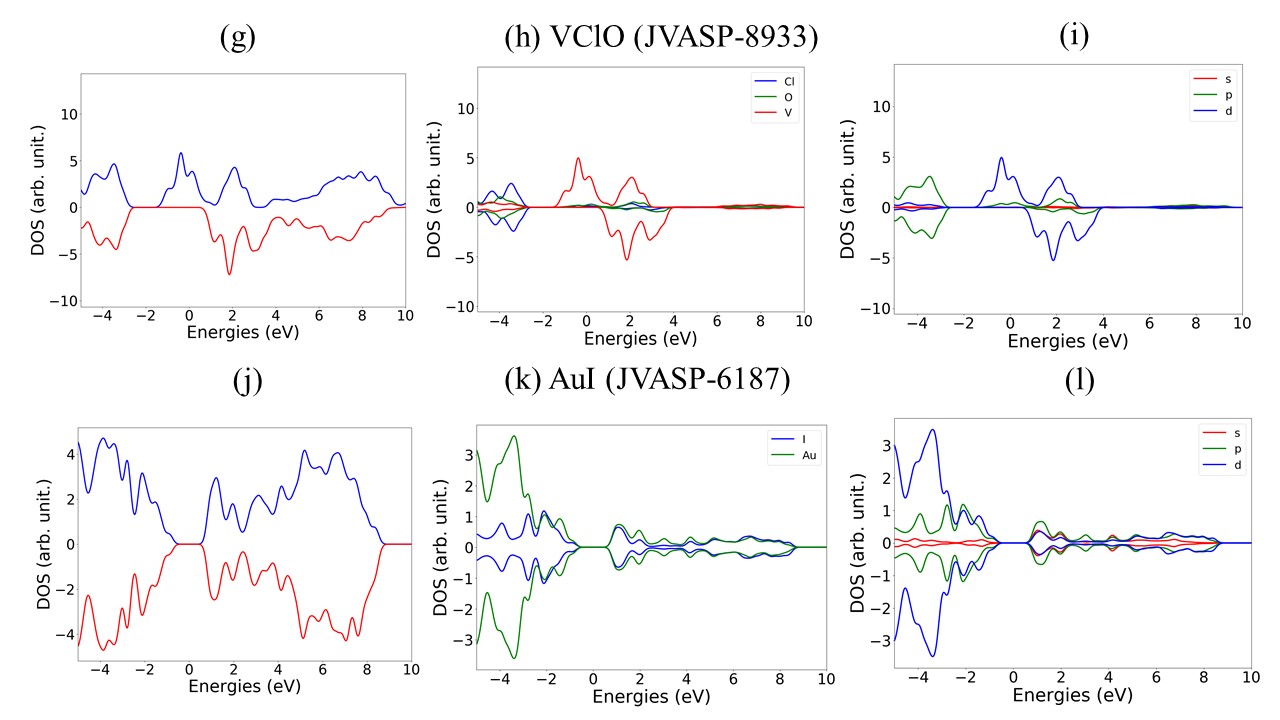


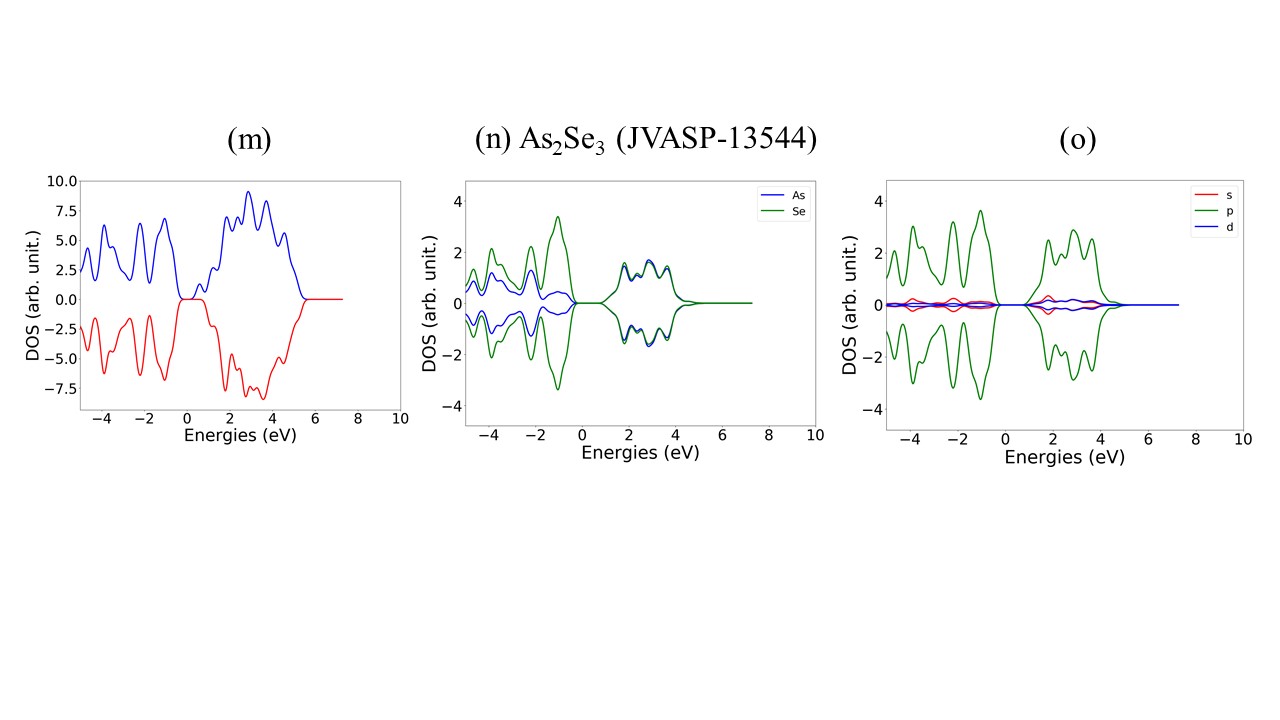


*Fig. S1 Total, element and orbital projected density of states of materials discussed in Fig. 4.*
